# An Observational Study to Analyse the Association of the ABO and Rh Blood Group Systems With Bronchial Asthma

**DOI:** 10.7759/cureus.37675

**Published:** 2023-04-17

**Authors:** Teena Lal, Muthukumar Sadhasivam, Sunitha Priya A K, Ajeet Arulkumar S J, Padmavathi R, Kouser Banu Khaleeluddin

**Affiliations:** 1 Physiology, Sri Ramachandra Institute of Higher Education and Research, Chennai, IND; 2 Physiology, Sri Muthukumaran Medical College Hospital & Research Institute, Chennai, IND; 3 Physiology, Mar Baselios Dental College, Ernakulam, IND; 4 Interventional Cardiology, Frontier Lifeline Hospital, Chennai, IND; 5 Clinical Skills, Imam Abdulrahman Bin Faisal University, Dammam, SAU

**Keywords:** rh-blood group antigens, immunology, hypersensitivity, abo blood-group antigens, bronchial asthma

## Abstract

Background

ABO blood group types are hereditary factors that have been shown to affect the blood levels of many antigens and proteins. Some blood groups have surprisingly been shown to be associated with some specific diseases, probably due to yet unexplained altering effects on the immune system or on the levels of other system-specific proteins. Studies conducted previously attempting to relate bronchial asthma and blood groups have had variable results and such studies on a large scale have not been ventured in India. Hence, the significance of the current study, which aims to search for an increased occurrence of bronchial asthma in any one of the ABO blood group phenotypes and additionally in Rh blood groups.

Objective

The objective of this study was to analyze the possible association of the ABO and Rh blood group types with bronchial asthma.

Methods

This is an observational study with 475 bronchial asthma patients, and 2052 non-asthmatic individuals belonging to the same geographical zone. After obtaining informed consent, ABO and Rh Blood group testing was done on the study subjects using the hemagglutination method. Chi-squared tests were employed for the comparison of proportions. Statistical significance was agreed upon for an error of 5%.

Results

The O blood group was predominant in both cases (46.9 %) and controls (36.1%). A chi-square analysis revealed a statistically significant higher percentage of O blood group in patients (χ2: 24.537; degrees of freedom (DF): 3; p-value = <0.01). The cases had more Rh -ve individuals (12%) than controls (8%), which too was statistically significant (χ2: 6.711; degrees of freedom (DF): 1; p-value = 0.01).

Conclusion

The present study shows a positive association between the O blood group and the Rh-negative blood group with bronchial asthma.

## Introduction

With the earliest recorded probable testimonial as “noisy breathing” dating back to 2600 BC (China) [1], bronchial asthma continues to be a problem in the modern world although today, we are much better equipped to deal with it. Being a chronic inflammatory condition of the airways, the cause of which is not well understood as yet, this leads to varying degrees of airway obstruction and wheezing. The shortness of breath, tightness in the chest, and mostly non-productive cough vary with time and severity, characterized by night-time flare-ups [2,3]. Genetic, environmental, and socioeconomic factors at large determine the occurrence of bronchial asthma [4-6].

Being one of the most common chronic respiratory diseases, bronchial asthma owns a heavy global burden of 358.2 million patients throughout the world [7]. The prevalence rate of asthma in India was 2.05% with an approximate patient burden of 17.23 million in 2011 [8]. Mortality due to asthma is less when compared to its associated morbidity and untoward economic implications. According to Masoli et al., besides being a major cause of poor quality of life, disability, loss of wages, and health resource utilization worldwide, one out of every 250 deaths globally is attributable to bronchial asthma [9].

From the time of the discovery of the major blood groups, many studies were undertaken to find the association of blood group phenotypes with different diseases. A positive link between peptic ulcer and blood group O as well as carcinoma of the stomach and blood group A has been well established by previous studies [10,11]. Blood group A was found to be associated with carcinoma of the urinary bladder, colon, uterus, salivary glands, etc. as well [12].

The ABO blood group antigens are oligosaccharide surface markers found attached to proteins or lipids on the surface of red blood cells. The antigens of the Rh blood group are proteins that form a core complex that is critical to the structure of the red blood cell membrane [13]. The A, B, and H antigens can also be secreted in body fluids, including saliva, tears, urine, etc. though not in all individuals. Accordingly, they are labeled as secretors and non-secretors, this occurrence being genetically controlled [14]. The secretor status of the individuals also has an impact on immunity against several organisms, as indicated by a study by Zorgani et al. [15].

Previous studies have also attempted to analyze the significance of the ABO blood group system with respect to bronchial asthma. While some studies did not show a significant association between bronchial asthma and blood groups [16], others, though showing significant association with any one of the blood groups, varied in different geographical areas [17,18].

Thus, this study aims to find out a possible association of any specific ABO and Rh blood groups with bronchial asthma by comparing the distribution of the blood group types in asthmatic patients with that of non-asthmatic controls from the same geographical region.

## Materials and methods

Approval from the institutional ethics committee of Sri Muthukumaran Medical College Hospital & Research Institute and informed consent from the study subjects were obtained before taking blood samples. The blood groups of 475 diagnosed cases of bronchial asthma patients from respiratory medicine camps, the outpatient department, casualty, and inpatients of Sri Muthukumaran Medical College Hospital and Research Institute, over a period of two years, were tested. The patients were selected from the age group of 18 to 62 years. This was done so as to include only the established cases of adult asthma with a typical history, and it is difficult for children to accurately follow the instructions of pulmonary function tests (PFTs). Among some older patients with suggestive history, those who were not able to do pulmonary function tests as per directions and some patients with ambiguous results of the test were also excluded.

According to the British Thoracic Society, the diagnosis of bronchial asthma has to be made based on the recognition of a characteristic pattern of respiratory symptoms, signs, and test results and the absence of any alternative explanation for these [19]. The current study followed the methodology advised by the British Thoracic Society to assess the initial probability of asthma by undertaking a structured clinical assessment. Adults with a typical clinical history, including recurrent episodes of symptoms (attacks), of a wheeze heard by a healthcare professional, a historical record of variable airflow obstruction, a positive history of atopy, and without any features to suggest an alternative diagnosis were categorized as having a high probability of asthma. Only patients with a high probability of asthma were selected for doing lung function tests using the RMS Helios 401 PC-based spirometer (Recorders & Medicare Systems Private Limited, Panchkula, Haryana, India) and patients showing an obstructive pattern with bronchodilator reversibility only were included as cases. PFT was not done for patients who had occasional isolated episodes of cough or breathlessness but without a history of characteristic symptoms of bronchial asthma and they were excluded from both cases and controls.

The blood groups of 2052 non-asthmatic subjects from the same geographical area, in the age range of 18 to 50 years, were selected from among the blood donors to the hospital as the control group. PFT was not done for these subjects, but a structured clinical assessment was done by means of a detailed medical history. To minimize errors, those who had any present or past history even remotely suggestive of asthma, like recurrent cough/breathlessness, etc., were totally excluded from this group.

Capillary blood was collected by the finger prick method. Blood grouping was done by the standard tile method with monoclonal antibodies (brand-Eryclone), anti-A, anti-B, and anti-Rh. The results were statistically analyzed by SPSS software version 24 (IBM Corp., Armonk, NY). Chi-squared tests were employed to contrast proportions and the odds ratio was calculated. A P-value of less than 0.05% was considered significant.

## Results

The study involved 475 bronchial asthma patients with 239 males and 236 females in the age range of 18-62 years. Nonasthmatic controls were 2052 in number, consisting of 1433 males and 619 females between the ages of 18 and 50. The ABO and Rh blood group distribution among cases and controls are shown in Table 1.

**Table 1:** Distribution of blood groups among cases and controls

ABO BLOOD GROUP	ABO blood group distribution				Total
	Asthmatics		Nonasthmatics		
	N	%	N	%	
A	111	23.4	479	23.3	590
AB	25	5.3	179	8.7	204
B	116	24.4	653	31.8	769
O	223	46.9	741	36.1	964
Total	475	100.0	2052	100.0	2527
Rh BLOOD GROUP	Rh blood group distribution				Total
	Asthmatics		Nonasthmatics		
	N	%	N	%	
Rh-Positive	418	88	1883	91.7	2301
Rh-Negative	57	12	169	8.2	226
Total	475	100	2052	100	2527

Of the 2052 controls, the O blood group was the predominant phenotype among the ABO blood groups, comprising 36% of the controls followed by B (32%), A (23%), and AB (9%). Among the patients with asthma, the O blood group also had the highest prevalence (47%) followed by B(24%), A (23%), and AB (5%). AB blood group had the least prevalence in both groups. A statistically significant difference was observed in the frequency of distribution of the ABO blood group between controls and asthmatics using the multinomial logistic regression. (χ2: 24.537; degrees of freedom (DF): 3; p-value = <0.01). The O blood group had a higher frequency in asthmatics than the controls, as shown in Table 2.

**Table 2:** Contingency table for the odd’s ratio of the O blood group among cases and controls OR=1.56, 95% CI (1.28-1.91), p < 0.0001

O blood group	Bronchial asthma	
	Yes	No
Yes	223	741
No	252	1311
Total	475	2052

The Rh blood group distribution had the following characteristics features: Rh positive was more common than Rh negative in both the cases and controls, as expected. The chi-square analysis showed that the cases had a statistically significantly higher proportion of the Rh-negative blood group compared to the controls (12% Vs 8%), as shown by the odds ratio calculated in Table 3.

**Table 3:** Contingency table for the odd’s ratio of Rh-negative blood groups among cases and controls OR=1.52, 95% CI (1.11-2.09), p=0.01

Rh-negative		Asthmatics	Nonasthmatics	Total
	Yes	57 (12%)	169 (8.2%)	226
	No	418 (88%)	1883(91.7%)	2301
Total		475	2052	2527

## Discussion

This study has shown a significantly higher frequency of both the O blood group and Rh-negative blood groups among patients with asthma than the controls. The result of the current study is comparable to the findings by Bijanzaadeh et al. who observed a significantly higher proportion of the O blood group among asthmatic patients followed by the B, A, and AB blood groups, respectively [16]. The current study is at least partially in agreement with a study done in Haryana, Northern India, which showed that the allele frequency of O was highest in asthmatics followed by B and A, though it did not find any difference in the Rh + phenotype between asthmatics and non-asthmatics [17]. According to a scoping review article including case-control studies, prospective or retrospective cohort studies, cross-sectional studies, and experimental studies, it was found that blood group O is prominent in patients with allergic rhinitis and asthma [20].

But the results are quite unlike the findings of Brachtel et al., who studied German patients experiencing atopic conditions (including atopic dermatitis, allergic rhinitis, and bronchial asthma) and noted a higher occurrence of subjects with blood group antigens A and B among patients than in controls without such diseases [21]. The above study included not only bronchial asthma but also included other related pathologies, like allergic rhinitis and atopic dermatitis, which may explain at least some of the differences in the results from the current study.

Bożena Mroczeket et al., in a retrospective study of bronchial asthma and COPD patients taken together, also concluded that the prevalence of such diseases is more in pooled non-O blood types (A, B, and AB) as compared with that in the O blood group [18]. A systematic review done by Samuel N Uwaezuoke et al. based on articles published within the past 45 years concluded that there is no unanimity on the specific histo-blood groups linked to respiratory atopy risk in the pediatric population [22]. A study by Moses Nnaemeka Alo, which attempted to find a relationship between ABO and Rhesus blood groups and susceptibility to asthma in Nigeria found no statistically significant difference between the Rh system in study subjects and controls, but blood group A was found to be significantly higher in asthma patients as compared to controls [23].

A study by Nelson et al. in Brazil has picked up a strong association between the O blood group and allergic rhinitis but only in males [24]. Allergic rhinitis and asthma are respiratory diseases having related immunopathological mechanisms, representing the phenotypic expression of one disorder with a wide spectrum of severity [25]. Hence, it is possible that the pathways involved in the relationship between the O blood group and allergic rhinitis also favor its connection with bronchial asthma. Different blood groups result in distinct cell membrane glycoconjugate complexes, which may possibly create diverse binding loci on the terminal structure of the oligosaccharide chains [26]. It is probable that these glycoconjugates, which act as prospective receptors for microbial or other extraneous antigens may bind allergens and affect the immune response variably in different blood groups, which might be the basis for increased frequency of bronchial asthma and certain other diseases in a particular blood group [27].

Limitations

The limitations of the study were as follows: 1) There is a lack of exact age and sex matching among cases and controls, as the controls were taken from among blood donors, which had a disproportionately higher male representation; 2) Controls were taken from blood donors in the hospital, which may mirror the transfusion requirements of the hospital and may not accurately represent the community distribution of blood groups; 3) The pulmonary function tests were not done for the control subjects to rule out bronchial asthma and only a structured clinical assessment with a detailed medical history was done. However, for minimizing errors, all subjects who had any present or past history even remotely suggestive of asthma, like recurrent cough/breathlessness, etc., were excluded from this group.

On the other hand, the strengths of the study were a bigger sample size and the same pattern of distribution of the ABO blood group among the two groups.

## Conclusions

From the results of the study, we observed that bronchial asthma is more prevalent in the O blood group and Rh-ve blood group phenotypes as compared with other blood groups. Respiratory mucosal evaluation and investigations at the genetic, antigenic, and molecular levels may throw further light on the influence of blood group antigens and their subtypes on chronic respiratory diseases. Future studies involving the genotypic distribution and quantitative assessment of the blood group antigens and their subtypes in asthmatic subjects may lead to new perspectives on the management of bronchial asthma.
